# Correlations between the EMG Structure of Movement Patterns and Activity of Postural Muscles in Able-Bodied and Wheelchair Fencers

**DOI:** 10.3390/s23010135

**Published:** 2022-12-23

**Authors:** Zbigniew Borysiuk, Monika Blaszczyszyn, Katarzyna Piechota, Mariusz Konieczny, Wojciech J. Cynarski

**Affiliations:** 1 Faculty of Physical Education and Physiotherapy, Opole University of Technology, 45-758 Opole, Poland; 2 Institute of Physical Culture Studies, College of Medical Sciences, University of Rzeszow, 35-959 Rzeszow, Poland

**Keywords:** electromyography, postural muscles, wheelchair fencing, heuristic analysis

## Abstract

The study involved Paralympic wheelchair fencers (N = 7) in two disability categories, and able-bodied female epee fencers (N = 7), members of the polish paralympic fencing teams. The performance of postural muscles and sword arm muscles in both groups of fencers, and of the front and rear leg muscles in the able-bodied fencers, was examined using surface electromyography with an accelerometer and the OptiTrack motion analysis system, as well as ground force reaction platforms. The activation sequence of individual muscles was determined and the structure of movement patterns in able-bodied and wheelchair fencers was formulated. A statistically significant correlation was found between the complex motor reaction time and latissimus dorsi muscle activation (*p* = 0.039, Z = −2.062) in wheelchair fencers. High correlations between the vertical force and EMG signal values of the gastrocnemius caput laterale muscle (0.85 for *p* = 0.022) were found in able-bodied fencers. A heuristic analysis indicated the significance of postural muscles in the movement patterns of wheelchair and able-bodied fencers. These muscles play a crucial role in the anticipatory postural adjustment of the trunk during technical fencing actions, including attacks on the opponent’s body.

## 1. Introduction

Among theories of human motor behavior, the most pivotal appears to be observations of Nikolai Bernstein included in his seminal work The Co-ordination and Regulation of Movements (Pergamon Press, Oxford, 1967) [1]. One of Bernstein’s most distinctive premises is the concept of “functionally freezing degrees of freedom”, which is of fundamental significance to motor learning. The improvement of motor activities with regard to productive motor skills, sports, or daily motor activities consists of the elimination of unnecessary muscle tensions based on motor feedback networks that interfere with coordinated actions. According to Richard A. Schmidt, all motor activities rely on generalized motor programs encoded in the motor areas of the cerebral cortex [2]. The motor programs become fixed through learning and training or fade away in accordance with synaptic plasticity mechanisms. While long-term potentiation strengthens synaptic connections, long-term depression contributes to the inactivation of neuronal networks [3] and the loss of unused neural connections.

The above premises lay the foundation for the control and regulation of human motor activities [4]. In modern times, they have contributed to the development of cybernetics and robotics. The resulting knowledge is the basis for the construction of robots and devices replacing human motor functions lost randomly or due to aging. For example, in the field of biomedical engineering, brain–computer interfaces are constructed using EMG signals to acquire, through electrodes, data that enable the control of prostheses [5,6].

In recent years, the interest and competitiveness in sports for people with disabilities have grown significantly, creating a need for technological tools supporting athletic performance. The most widely used technologies have been inertial and EMG sensors and wheelchair sports have been studied most frequently. In research using EMG sensors, motion trackers have been fundamental to recognizing muscle activation patterns, providing kinematic information on temporal occurrences, and analyzing the recorded EMG signal. These technologies are used to enhance elite and amateur sports training for able-bodied and disabled athletes [7,8,9].

Technical measures providing quantitative information about an athlete’s technique are fundamental to enhancing the correct execution of movement and to injury avoidance [10].

Both the analysis of muscle tension structure measured with EMG and the recording of postural muscle using ground reaction force platforms play an extremely important role in human motor activity research. The premise here is that any motor activity must be preceded by an anticipatory postural adjustment mechanism activating the postural muscles, which ensures the correctness of movement patterns [11]. The importance of this process has been confirmed in studies of wheelchair athletes whose dorsal and abdominal postural muscles are shown to be activated first [12,13,14]. The dysfunctions and reduced psychomotor performance of wheelchair athletes induce compensatory and adaptive neurophysiological processes, which promote the formation of proper movement patterns ensuring the effectiveness of sports technique. It should be noted that athletes with disabilities such as amputations or paraplegia demonstrate, in general, similar movement patterns to their able-bodied counterparts. This shows that teaching and improving movement programs at the central nervous system level take place in similar neuro-psychological conditions [15,16,17].

The participants in the present study were wheelchair and able-bodied fencers. Both groups represented a high level of sportsmanship and were members of the polish paralympic fencing teams, respectively.

The main hypothesis of the study was that wheelchair fencers featured similar movement patterns while executing a lunge attack to the opponent’s torso to able-bodied fencers. Despite the significant dysfunctions of fencers with disabilities, the anticipatory activation in their movement structure involves the dorsal and abdominal muscles as postural muscles. With regard to able-bodied fencers, an analogous function is performed by the gastrocnemius muscles of the lower leg, in particular, the rear leg [18,19].

Long-term research on wheelchair fencers and able-bodied fencers has made it possible to accurately identify their model movement characteristics. The present study used the EMG signal analysis of generated bioelectric stimulation expressed in microvolts [μV] and the time of activation of individual muscles expressed in milliseconds [ms]. In the case of able-bodied fencers, a ground force reaction platform was also used to measure the vertical and horizontal forces of the activated muscles of both legs. All studied motor actions were triggered by signals given by the coaches participating in the experiment [20,21].

## 2. Materials and Methods

**Figure 1:**
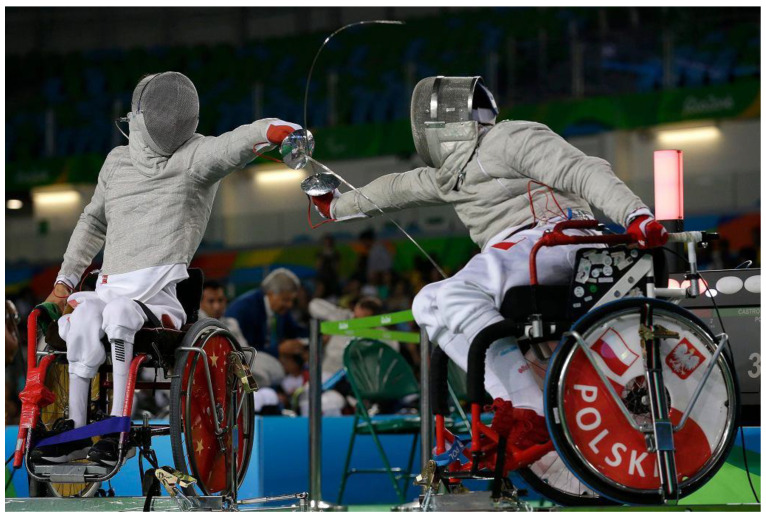
Wheelchair fencing medalist Paralympic Games.

The participants included 7 male and female wheelchair fencers and 7 female able-bodied fencers. The wheelchair fencers belonged to two disability categories: Category A—amputees or individuals with mild paralysis of the lower limbs; and Category B—athletes with spinal cord injuries and paraplegia. As members of the Paralympic team, the fencers represented a high international level. The able-bodied female fencers were 7 members of the Poland national epee fencing team, including the world individual and team vice-champions (Figure 1). Surface electromyography (sEMG) was used to measure muscle activity in both wheelchair and able-bodied fencers. To determine the time from stimulus onset to muscle bioelectrical activation, an accelerometer was used for the wheelchair fencers and OptiTrack markers for the able-bodied fencers. Due to the lower limb dysfunctions of the wheelchair fencers, it was not possible to use ground force reaction platforms with them, but only with the able-bodied fencers.

A 16-channel EMG system (Noraxon, DTS, Desktop Direct Transmission System, Scottsdale, AZ, USA) was used for the study with a sampling frequency of 1500 Hz and a resolution of 16 bits with dedicated data analysis software (MyoResearch XP Master Edition for DTS Noraxon, Opole, Poland). A wireless transmitter-recorder was used to synchronize the EMG system and transfer the EMG signal directly to the computer (3-axis wireless DTS 3D accelerometer sensor with ±6 g nominal output range, ±0.67 V/g sensitivity, and 5 Hz–1.8 kHz bandwidth). The sEMG signals were smoothed by estimating the square root mean in a window of 300ms. All signals were normalized in reference to these values and expressed as a percent. The research procedure used in the study was the same as in the entire project and previous articles [12,13].

**Figure 2:**
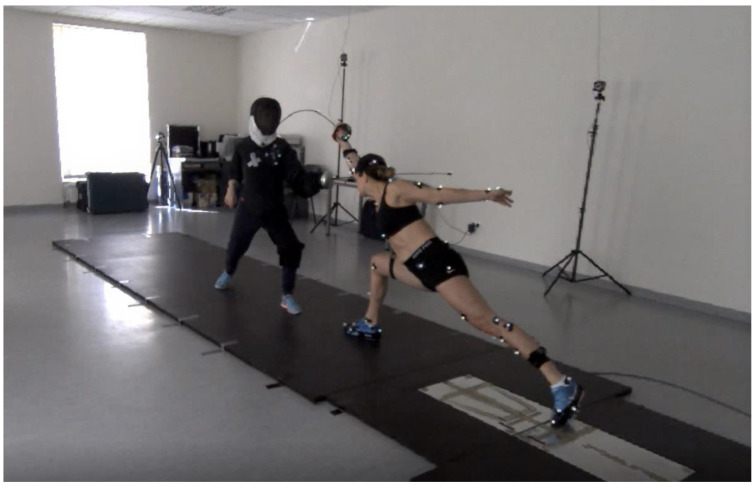
A lunge executed by the saber fencing world champion (Diagnostic Laboratory of the Department of Human Movements, Opole University of Technology).

Both able-bodied and wheelchair fencers underwent similar testing procedures. The coach initiated three attempts of direct thrusts to a visual stimulus (coach’s blade movement from parry quarte to parry sixte) (Figure 2). The attempts were preceded by a 20-min warm-up. The EMG electrodes were placed on selected muscles of the fencers just before the warm-up to avoid their detachment during the exercise.

## 3. Statistical Analysis

The obtained data were processed using Statistica 13.1 (StatSoft, Inc., Oklahoma, OK, USA). The hypothesis was verified at the significance level of *p* ≤ 0.05. The assumption of a normal distribution of analyzed statistical data was checked with the Shapiro–Wilk test.

Since not all the statistics met the assumptions of normal distribution, the nonparametric Spearman’s rank correlation coefficient (R) and the Wald–Wolfowitz runs test were applied.

## 4. Results

### 4.1. Wheelchair Fencers

Data from Table 1 revealed a statistically significant difference between the two categories of fencers in terms of complex reaction time representing the activation of the LD LT muscle (*p* = 0.039, Z = −2.062).

### 4.2. Able-Bodied Fencers

**Figure 3:**
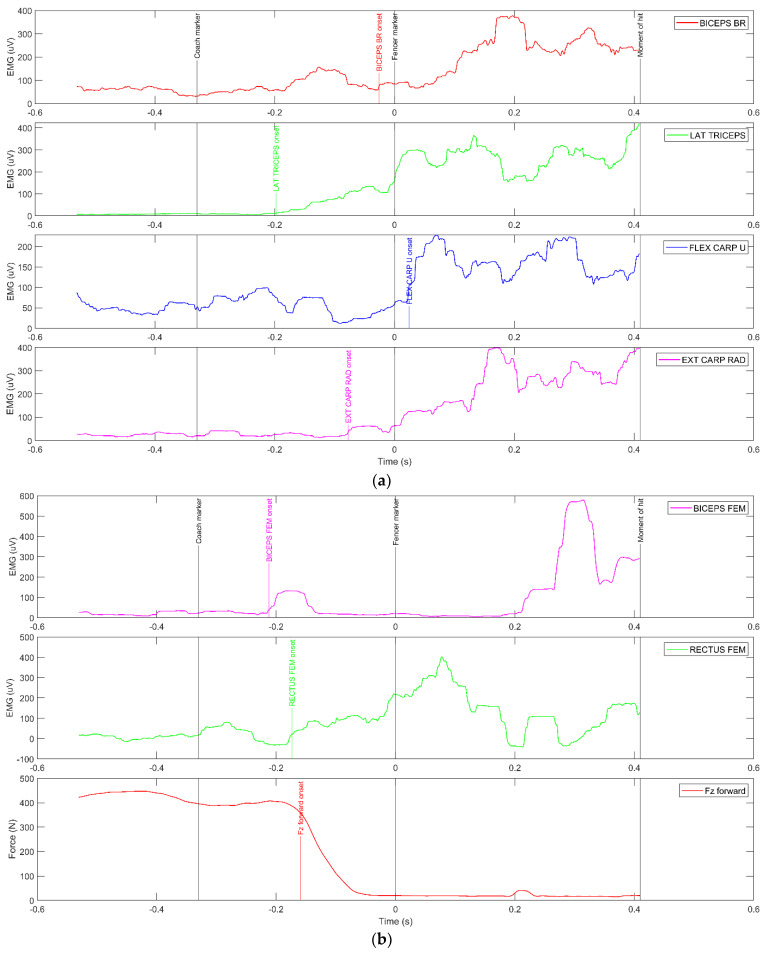

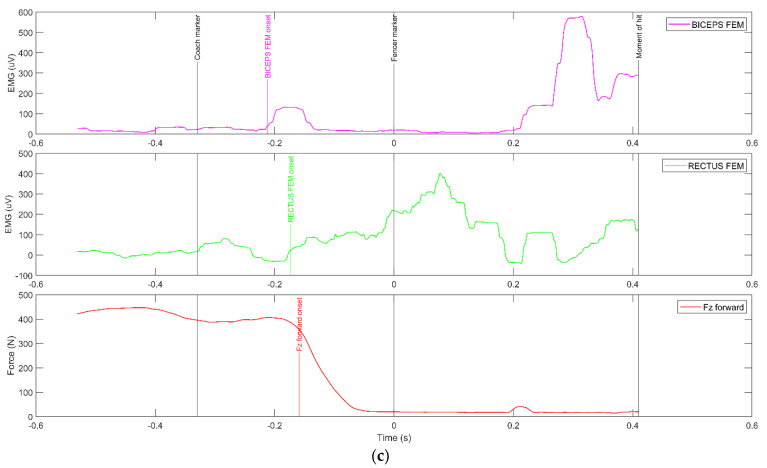
(**a**) Waveforms of muscle activity and ground reaction forces with marked moments of coach’s movement (Coach markers)—arm muscles (FCU, ECR, BB, TB). (**b**) Waveforms of muscle activity and ground reaction forces with marked moments of coach’s movement (Coach markers)—rear leg muscles (RF, BF). (**c**) Waveforms of muscle activity and ground reaction forces with marked moments of coach’s and fencer’s movement (Coach markers and Fencer markers)—front leg muscles (RF, BF).

Figure 3a,b [22] present waveforms of muscle activity and ground reaction forces with marked moments of the coach’s movement (Coach markers) of arm and leg muscles present. The onset of muscle activity (above the mean value + 3SD calculated from 100ms after assuming an appropriate fencing pose) and the onset of the fencer’s movement from the marker on the hand (MATLAB findchangepts automatic function) (fencer marker) to perform the push are displayed.

The analysis of the muscle activation sequence in Figure 3a,b shows a clear trend of anticipatory activation of the LAT GAS and MED GAS muscles of the rear leg. Only slightly later is the TB muscle activated, followed by the ECR, BB, and FCU muscles. Characteristically, the arm and forearm extensors are activated. More importantly, there is a specific synergy of the gastrocnemius muscles with the vertical ground forces. During the movement phase, the FZ rear is approximately 600 N and the MED GAS is approximately 100 μV. During the actual movement time, the FZ rear was at approximately 800 N max, while the EMG was approximately 250 μV max relative to the LAT GAS.

When performing a lunge, the coordinated activity of all limbs can be observed, with the non-attacking arm playing the smallest part since it only assists in maintaining balance after a hit is placed on the opponent’s body. The main importance is attributed to the rear leg and the dominant sword arm. As shown in Figure 3b,c, the RF and BF muscles of the front leg are activated later than the muscles of the rear leg; however, in a certain synergy with the peak of the vertical force Fz for the front leg. During the dynamic straightening of the rear leg, the front leg is lifted off the ground, and during extension, the RF muscle (approximately 400 μV) is first activated as an extensor followed by the BF muscle (approximately 800 μV) just before the hit. Characteristically, both the RF and BF muscles reach their activation peaks following the fencer’s blade movement (Fencer marker). At this point, the Fz force of the front leg fades because just before the fencer’s blade movement the fencer’s front leg leaves the ground force reaction platform. It should be noted, as shown in Figure 3c, that the fencer’s front foot presses the platform anticipatively while still in the pre-motor phase of performing the lunge, which generates a significant amount of vertical force.

The vertical force waveforms of the rear leg (Table 2) revealed high correlations with the EMG waveforms of the GAS muscles with small time shifts relative to each other (in most cases, a delay in force versus EMG of approximately 15 ms). The exception was Fencer 6, in whom the correlations were moderately negative and the recorded times were long.

## 5. Discussion and Conclusions

The study revealed that during a lunge attack on the coach’s torso, the wheelchair fencers first activated their dorsal muscles, followed by the deltoid muscle of the dominant sword arm and the abdominal muscles. At the end of the attack phase, activity was recorded in the forearm muscles and arm extensors. The entire movement pattern closed with the activation of the BC RT FCR flexors. It can be concluded that the postural muscles are activated first or in synergy with the arm extensors, which, in accordance with the definition of attack in fencing should initiate an offensive action [23,24].

The extraordinary mobility of the trunk and arms during wheelchair fencing combat results in imbalances. In order to maintain a stable posture, a wheelchair fencer’s central nervous system triggers an anticipatory mechanism that activates the postural muscles. A similar phenomenon is observed in able-bodied fencers. The analogy here is the anticipatory muscle activation (from tens to approximately 150 ms) of the rear leg, in particular, of the gastrocnemius muscle while executing a lunge [25,26]. The phenomenon of anticipatory activation is intended not only to maintain the fencer’s stable posture in the wheelchair but also to quickly move the torso toward the target area following the attacking arm. The effect of this is to reduce the complex reaction time and movement time in response to the opponent’s actions [20,27].

As reported by previous researchers, the EMG signal (% MVC) should correlate with reaction time, in particular, movement time and complex motor reaction in both wheelchair and able-bodied fencers [28]. In this case, the conclusion on the mobilization of more motor units affecting the level of MVC is legitimate.

With regard to able-bodied fencers, following the main assumption of the study, three muscles play a key role in the movement phase, i.e., the gastrocnemius muscle of the lower leg and the arm extensors. In synergy with the vertical force of the rear foot, these muscles can be treated as indicators of the activity of the muscular system during the execution of a lunge. A noteworthy part of the analysis is the fact that at the moment of hit, after more than 400 ms from the onset of the visual stimulus to the commencement of the action, bioelectrical activity at more than 400 [μV] is indicated in four muscles of the attacking arm, with a lower proportion of approximately 250 [μV] in the BB muscle. At the moment of the hit, the force indicators of both the front and rear legs practically fade away [29].

Using interdependence analysis and considering the responses to the visual stimulus, the wheelchair fencers only demonstrated a high correlation coefficient (r = −0.821) between their complex reaction time (CRT) and the EMG %MVC signal value of the EAR RT muscle. This is rather significant as it shows that the muscles reducing the CRT, i.e., muscles affecting the attacking efficiency of wheelchair fencers, were the external abdominal oblique and latissimus dorsi postural muscles [30].

Addressing the core hypothesis of the present study, the heuristic analysis proved that, despite differences in the research methodology between wheelchair fencers and able-bodied fencers, both groups of fencers share their movement patterns as described by the timing and EMG signals. The decisive role in their movement structure is played by postural muscles, which preemptively stabilize the trunk during attacks in both groups of fencers. This is of fundamental importance due to the extraordinary dynamics of technical actions during fencing competitions. The similar structure of motor programs at the level of the motor cortex points to the importance of postural muscles within the mechanism of anticipatory postural adjustment. Considering this phenomenon in the training process can ensure efficiency in terms of speed and accuracy of technical coordination activities while preventing undesirable injuries and traumas [31,32]. The consideration of the premise of “functionally freezing degrees of freedom” makes it possible to understand which muscles and in what activation sequence determine the efficiency of movement patterns.

EMG analysis provides input for quantifying muscle effort by correcting and integrating the signal or calculating peak amplitude and for identifying specific muscle activation patterns and synergies as defined by the onset and offset of muscle activation. EMG analysis is commonly performed in sports to assess the amplitude of athletes’ muscle activation or to detect the onset and shifts of EMG activity; in other cases, frequency analysis allows the estimation of muscle fatigue [33].

In a number of studies, EMG was used to identify neuromuscular strategies adopted for task coping. Researchers found a significant difference in force generation with similar levels of EMG activity between able-bodied and disabled participants [34,35].

Teles et al. noted that indicators of static and dynamic strength were similar in Paralympic powerlifting triathletes with spinal cord injuries and other disabilities [36]. In turn, Nemet et al., when comparing able-bodied and disabled athletes, noted that their knee and trunk movements and muscle actions differed significantly [37].

The common denominator in studies on the classification of athletes with disabilities is the role of the trunk in sports performance as it plays an important role in the generation and transmission of force during various sports activities. In fact, the motor behavior of the trunk can be easily assessed using EMG sensors evaluating muscle activity related to trunk stabilization [35,38,39,40].

## Figures and Tables

**Table 1:** Statistical analysis (Wald–Wolfowitz runs test) based on the comparison of the mean time of all attempts performed by selected muscle groups in response to a visual stimulus in category A and B fencers [12].

Muscle	Variable	Wald-Wolfowitz Runs Test			
		Mean A Time (s)	Mean B Time (s)	Z	*p*-Value
DEL RT	Time (ms)	0.489	0.540	1.334	0.182
TRI RT	Time (ms)	0.560	0.575	0.485	0.628
ECR RT	Time (ms)	0.380	0.527	1.334	0.182
LD RT	Time (ms)	0.420	0.562	−0.364	0.716
LD LT	Time (ms)	0.333	0.522	−2.062	0.039
BC RT	Time (ms)	0.547	0.520	0.485	0.628
FCR RT	Time (ms)	0.617	0.631	0.485	0.628
EAO RT	Time (ms)	0.408	0.489	−0.364	0.716
EAO LT	Time (ms)	0.435	0.456	0.485	0.628

**Table 2:** Maximum mutual correlations of the waveforms of the rear leg forces and the EMG of the GAS muscle (in parentheses, the corresponding delay/anticipation times; positive values indicate the delay of the force in relation to EMG) [22].

	LAT GAS	MED GAS
Fencer 1	0.74 (0)	0.66 (0.013)
Fencer 2	0.86 (0.017)	0.87 (0.025)
Fencer 3	0.75 (−0.002)	0.67 (0.022)
Fencer 4	0.85 (0.022)	0.81 (0.030)
Fencer 5	0.85 (0.031)	0.79 (0)
Fencer 6	−0.51 (0.473)	−0.52 (0.462)
Fencer 7	0.61 (−0.013)	0.74 (0.011)

Note: All correlations are statistically significant (*p* < 0.05).

## Data Availability

Samples of the compounds are available from the authors upon written request.

## Abbreviations

RT	right side
LT	left side
DEL	deltoideus middle head
TRI	triceps brachii
BC	biceps brachii
ECR	extensor carpi radialis longus
FCR	flexor carpi radialis
LD	latissimus dorsi
EAO	external abdominal oblique
x, y, z	channel–accelerometer in 3 axes
GRF	ground reaction forces
Fz rear	vertical force of the rear foot
CRT	complex reaction time
MVC	maximal voluntary contraction
LAT GAS	gastrocnemius caput laterale
MED GAS	gastrocnemius caput mediale
ECR	extensor carpi radialis
FCU	flexor carpi ulnaris
BB	biceps brachii
TB	triceps brachii (lateral capture)
RF	rectus femoris
BF	biceps femoris
